# Effect of an Educational Toolkit on Quality of Care: A Pragmatic Cluster Randomized Trial

**DOI:** 10.1371/journal.pmed.1001588

**Published:** 2014-02-04

**Authors:** Baiju R. Shah, Onil Bhattacharyya, Catherine H. Y. Yu, Muhammad M. Mamdani, Janet A. Parsons, Sharon E. Straus, Merrick Zwarenstein

**Affiliations:** 1 University of Toronto, Toronto, Ontario, Canada; 2 Institute for Clinical Evaluative Sciences, Toronto, Ontario, Canada; 3 Sunnybrook Health Sciences Centre, Toronto, Ontario, Canada; 4 Li Ka Shing Knowledge Institute, St Michael's Hospital, Toronto, Ontario, Canada; 5 University of Western Ontario, London, Ontario, Canada; University of Glasgow, United Kingdom

## Abstract

**Background:**

Printed educational materials for clinician education are one of the most commonly used approaches for quality improvement. The objective of this pragmatic cluster randomized trial was to evaluate the effectiveness of an educational toolkit focusing on cardiovascular disease screening and risk reduction in people with diabetes.

**Methods and Findings:**

All 933,789 people aged ≥40 years with diagnosed diabetes in Ontario, Canada were studied using population-level administrative databases, with additional clinical outcome data collected from a random sample of 1,592 high risk patients. Family practices were randomly assigned to receive the educational toolkit in June 2009 (intervention group) or May 2010 (control group). The primary outcome in the administrative data study, death or non-fatal myocardial infarction, occurred in 11,736 (2.5%) patients in the intervention group and 11,536 (2.5%) in the control group (*p* = 0.77). The primary outcome in the clinical data study, use of a statin, occurred in 700 (88.1%) patients in the intervention group and 725 (90.1%) in the control group (*p* = 0.26). Pre-specified secondary outcomes, including other clinical events, processes of care, and measures of risk factor control, were also not improved by the intervention. A limitation is the high baseline rate of statin prescribing in this population.

**Conclusions:**

The educational toolkit did not improve quality of care or cardiovascular outcomes in a population with diabetes. Despite being relatively easy and inexpensive to implement, printed educational materials were not effective. The study highlights the need for a rigorous and scientifically based approach to the development, dissemination, and evaluation of quality improvement interventions.

**Trial Registration:**

http://www.ClinicalTrials.gov NCT01411865 and NCT01026688

*Please see later in the article for the Editors' Summary*

## Abstract 2

In a pragmatic cluster-randomized trial, Baiju Shah and colleagues evaluated the effectiveness of printed educational materials for clinician education focusing on cardiovascular disease screening and risk reduction in people with diabetes.

*Please see later in the article for the Editors' Summary*

## Editors' Summary

**Background:**

Clinical practice guidelines help health care providers deliver the best care to patients by combining all the evidence on disease management into specific recommendations for care. However, the implementation of evidence-based guidelines is often far from perfect. Take the example of diabetes. This common chronic disease, which is characterized by high levels of sugar (glucose) in the blood, impairs the quality of life of patients and shortens life expectancy by increasing the risk of cardiovascular diseases (conditions that affect the heart and circulation) and other life-threatening conditions. Patients need complex care to manage the multiple risk factors (high blood sugar, high blood pressure, high levels of fat in the blood) that are associated with the long-term complications of diabetes, and they need to be regularly screened and treated for these complications. Clinical practice guidelines for diabetes provide recommendations on screening and diagnosis, drug treatment, and cardiovascular disease risk reduction, and on helping patients self-manage their disease. Unfortunately, the care delivered to patients with diabetes frequently fails to meet the standards laid down in these guidelines.

**Why Was This Study Done?:**

How can guideline adherence and the quality of care provided to patients be improved? A common approach is to send printed educational materials to clinicians. For example, when the Canadian Diabetes Association (CDA) updated its clinical practice guidelines in 2008, it mailed educational toolkits that contained brochures and other printed materials targeting key themes from the guidelines to family physicians. In this pragmatic cluster randomized trial, the researchers investigate the effect of the CDA educational toolkit that targeted cardiovascular disease screening and treatment on the quality of care of people with diabetes. A pragmatic trial asks whether an intervention works under real-life conditions and whether it works in terms that matter to the patient; a cluster randomized trial randomly assigns groups of people to receive alternative interventions and compares outcomes in the differently treated “clusters.”

**What Did the Researchers Do and Find?:**

The researchers randomly assigned family practices in Ontario, Canada to receive the educational toolkit in June 2009 (intervention group) or in May 2010 (control group). They examined outcomes between July 2009 and April 2010 in all patients with diabetes in Ontario aged over 40 years (933,789 people) using population-level administrative data. In Canada, administrative databases record the personal details of people registered with provincial health plans, information on hospital visits and prescriptions, and physician service claims for consultations, assessments, and diagnostic and therapeutic procedures. They also examined clinical outcome data from a random sample of 1,592 patients at high risk of cardiovascular complications. In the administrative data study, death or non-fatal heart attack (the primary outcome) occurred in about 11,500 patients in both the intervention and control group. In the clinical data study, the primary outcome―use of a statin to lower blood fat levels―occurred in about 700 patients in both study groups. Secondary outcomes, including other clinical events, processes of care, and measures of risk factor control were also not improved by the intervention. Indeed, in the administrative data study, some processes of care outcomes related to screening for heart disease were statistically significantly worse in the intervention group than in the control group, and in the clinical data study, fewer patients in the intervention group reached blood pressure targets than in the control group.

**What Do These Findings Mean?:**

These findings suggest that the CDA cardiovascular diseases educational toolkit did not improve quality of care or cardiovascular outcomes in a population with diabetes. Indeed, the toolkit may have led to worsening in some secondary outcomes although, because numerous secondary outcomes were examined, this may be a chance finding. Limitations of the study include its length, which may have been too short to see an effect of the intervention on clinical outcomes, and the possibility of a ceiling effect—the control group in the clinical data study generally had good care, which left little room for improvement of the quality of care in the intervention group. Overall, however, these findings suggest that printed educational materials may not be an effective way to improve the quality of care for patients with diabetes and other complex conditions and highlight the need for a rigorous, scientific approach to the development, dissemination, and evaluation of quality improvement interventions.

**Additional Information:**

Please access these websites via the online version of this summary at http://dx.doi.org/10.1371/journal.pmed.1001588.

- The US National Diabetes Information Clearinghouse provides information about diabetes for patients, health care professionals, and the general public (in English and Spanish)
- The UK National Health Service Choices website provides information (including some personal stories) for patients and carers about type 2 diabetes, the commonest form of diabetes
- The Canadian Diabetes Association also provides information about diabetes for patients (including some personal stories about living with diabetes) and health care professionals; its latest clinical practice guidelines are available on its website
- The UK National Institute for Health and Care Excellence provides general information about clinical guidelines and about health care quality standards in the UK
- The US Agency for Healthcare Research and Quality aims to improve the quality, safety, efficiency, and effectiveness of health care for all Americans (information in English and Spanish); the US National Guideline Clearinghouse is a searchable database of clinical practice guidelines
- The International Diabetes Federation provides information about diabetes for patients and health care professionals, along with international statistics on the burden of diabetes

## Introduction

Diabetes is a common and serious chronic disease associated with impaired quality of life, premature mortality, and significant economic costs [1]–[3]. Patients with diabetes require complex care to manage multiple risk factors such glycemia, blood pressure, and lipids, and to screen for and treat the many complications of the disease. Clinical practice guidelines assist health care providers to deliver this complex care by synthesizing the enormous literature on diabetes management into specific recommendations for care [4]–[6]. However, gaps between guideline recommendations and actual care delivered to patients with diabetes are well documented [7]–[10]. Therefore, the implementation of evidence-based guideline recommendations needs to be improved.

Although multifaceted and intensive quality improvement interventions have been shown to improve processes of care for diabetes patients [11]–[14], they are often difficult to widely implement because of their complexity and cost. In contrast, printed educational materials for clinician education are one of the most commonly used approaches for quality improvement and for increasing guideline adherence, because they are familiar, relatively inexpensive, and easily scalable to reach large populations [15]. A systematic review of methods to improve guideline adherence found that dissemination of educational materials led to an absolute improvement of more than 8% for dichotomous outcomes [15]. Therefore, when the Canadian Diabetes Association (CDA) updated their national clinical practice guidelines for diabetes in December 2008 [5], they created a quality improvement strategy of mailed educational toolkits to family physicians targeting a sequence of key themes from the guidelines. The first theme was cardiovascular disease screening and treatment, since it is the most prevalent complication of the disease and since evidence-based preventative interventions are readily available. The objective of this study was to evaluate the effectiveness of this educational toolkit to improve management of cardiovascular risk factors and outcomes of cardiovascular disease in people with diabetes.

## Methods

We conducted a pragmatic cluster randomized controlled trial. A detailed description of the intervention and allocation has been previously published (Text S1) [16].

### Ethics Statement

The study was approved by the research ethics board of Sunnybrook Health Sciences Centre, Toronto, Ontario.

### Intervention and Allocation

The cardiovascular disease toolkit was a collection of printed educational materials, packaged in a brightly colored box with CDA branding, sent to Canadian family physicians. The contents included an introductory letter from the chair of the practice guidelines' Dissemination and Implementation Committee; an eight-page summary of selected sections of the practice guidelines targeted towards family physicians; a four-page synopsis of the key guideline elements pertaining to cardiovascular disease risk; a small double-sided laminated card with a simplified algorithm for cardiovascular risk assessment, vascular protection strategies, and screening for cardiovascular disease; and a pad of tear-off sheets for patients with a cardiovascular risk self-assessment tool and a list of recommended risk reduction strategies [16]. The toolkit was created for the CDA by clinical experts including family physicians, endocrinologists, and other health care professionals, with guidance from clinicians with expertise in knowledge translation and implementation. The implicit theory behind its development was that the guidelines were too long and complex to be easily incorporated into clinical practice, so the toolkit aimed to simplify the information, tailor it towards clinical practice, and provide explicit actionable recommendations.

While the toolkit was sent to most family physicians in Canada, family practices in the province of Ontario were allocated 1∶1 into the intervention or control group using random number sequences generated by SAS version 9.3 (SAS Institute Inc.), stratified by the 14 health regions into which responsibility for health care delivery in Ontario is divided. Randomization at the practice level helped prevent contamination by ensuring that all patients seen at a single location were assigned to the same study arm. An independent analyst, not otherwise involved with the study, generated the randomized list and provided it to the mailing house distributing the toolkit on behalf of the CDA. The toolkit was mailed in June 2009 to all family practices assigned to the intervention arm, and in May 2010 to practices assigned to the control arm. Physicians were unaware that they were part of a randomized trial.

### Data Sources and Study Participants

Two separate studies were conducted. Both studies were registered after trial initiation. In early 2009, the randomization process was conducted to assign family practices to receive the toolkit early (June 2009) or late (May 2010), in the anticipation of acquiring future funding to evaluate the effectiveness of the intervention. Such funding was received in October 2009, and the clinical data study was registered in December 2009, prior to the recruitment of family practices and patients to participate in the study. The administrative data study was registered in August 2011, linked to the earlier registration for the clinical data study. It used data that were routinely collected by the Ministry of Health for the administration of the health care system, not specifically for the purposes of research nor for this study. Analyses of the administrative data were only conducted once these data were received and linked in early 2012.

#### Administrative data study

A province-wide study was conducted using population-level health care administrative data from the Ontario Ministry of Health and Long-Term Care. Because of the single payer health care system in Ontario, these data cover the entire population with no loss to follow-up. These data are collected routinely for health care administration purposes, independently of this trial. Available data sources included a demographic database; records of all hospital separations and emergency department visits; physician service claims for consultations, assessments, and diagnostic and therapeutic procedures; and prescriptions filled under the provincial formulary, which provides universal drug insurance coverage for all residents aged ≥65 years. Individuals are linked between all of these databases and across time using a unique anonymous identifier.

The study included all residents of Ontario aged ≥40 years who were diagnosed with diabetes as of 1 July 2009. Individuals with diabetes were identified using the Ontario Diabetes Database, a registry of all people with diagnosed diabetes derived from the administrative databases [17]. The ODD has a sensitivity of at least 86% and specificity of 97% when compared to primary care chart abstraction [17]. Residents of long-term care facilities were excluded. Each patient was assigned to their regular family practice [18]. Individuals who could not be assigned to a family practice were excluded.

#### Clinical data study

Because many important measures of quality of diabetes care are not available from administrative databases, we conducted a separate study in which detailed clinical data were directly collected. We randomly selected practices from each of the intervention and control arms, and randomly selected one physician from each practice. For practical reasons, recruited practices were within 150 km of the study center (67% of all practices in Ontario were within this radius). Each selected physician was contacted, and if willing to participate in the study, we randomly selected 20 diabetic patients who had visited the physician during the study period, and who fulfilled the CDA's definition of being at “high risk for cardiovascular events” [5]: (i) men aged ≥45 years, women aged ≥50 years, or (ii) men aged <45 years and women aged <50 years with at least one of: macrovascular disease; microvascular disease; a history of premature coronary or cerebrovascular disease in a first-degree relative; LDL cholesterol >5.0 mmol/l; systolic BP >180 mmHg; or duration of diabetes >15 years with age >30 years.

Patients were selected using random number sequences generated by SAS version 9.3 (SAS Institute). Their charts were reviewed by a trained and experienced registered nurse, blinded to treatment allocation, who abstracted relevant data into a computerized data collection template. Data elements collected included demographic information, medical history, medication utilization, blood pressure and anthropometric measurements, and laboratory test results.

### Outcomes

All patients were followed up for ten months, between July 2009 and April 2010 (i.e., the period when only the intervention group had received the toolkit). Outcomes were ascertained at the patient level.

#### Administrative data study

The primary outcome was the composite endpoint of death or non-fatal myocardial infarction. Secondary clinical event outcomes included all-cause death; myocardial infarction; myocardial infarction or unstable angina; stroke; stroke or transient ischemic attack; the composite of death, non-fatal myocardial infarction or non-fatal stroke; and the composite of death, non-fatal myocardial infarction, non-fatal stroke, unstable angina, or transient ischemic attack. Because the toolkit particularly highlighted new guideline recommendations on the use of diagnostic testing for coronary artery disease, process measures of care related to this testing were examined, included electrocardiograms, cardiac stress tests or nuclear imaging, coronary angiography, and ambulatory cardiology or internal medicine visits. For patients who were at least 65 years of age and therefore eligible for the provincially funded drug insurance program, drug prescriptions outcomes for cardiovascular risk reduction and treatment were also examined: initiation of an angiotensin-converting enzyme inhibitor or angiotensin receptor blocker; initiation of at least one anti-hypertensive from any class; initiation of medications from at least two anti-hypertensive classes; initiation of medications from at least three antihypertensive classes; initiation of a statin; initiation of any glucose-lowering drug; initiation of insulin; and initiation of nitrates. Prior prescriptions in the previous 10 months were used to separate ongoing prescriptions from initiation.

#### Clinical data study

In the clinical data study, outcomes were related to cardiovascular risk reduction. The primary outcome was the use (initiation or ongoing) of a statin during the observation period of July 2009 to April 2010. Secondary outcomes included: use of an angiotensin converting enzyme inhibitor or angiotensin receptor blocker; HbA1c≤7.0%; blood pressure ≤130/80; LDL cholesterol ≤2.0 mmol/l; and total- to HDL-cholesterol ratio ≤4.0. We also examined “clinical inertia” outcomes, where a measurement clearly above established treatment targets did not elicit a change in the patient's treatment at either the current or subsequent visit [19]. We examined changes in glucose-lowering treatments following an HbA1c >8.0%; changes in blood pressure-lowering treatment following a blood pressure >140/90; and changes in lipid-lowering treatment following an LDL cholesterol >3.0 mmol/l. In all cases, either pharmacological or non-pharmacological treatment changes were considered as absence of clinical inertia.

### Analysis

The statistical analysis was completed on an intention to treat basis. The analysis accounted for the cluster randomized design by using logistic regression models estimated using generalized estimating equation (GEE) methods to assess the statistical significance of the intervention's effect on each outcome. Explanatory variables in all models were the randomization arm (the variable of interest) and diabetes patient volume as practice-level variables, and age, sex, diabetes duration, and previous cardiovascular disease (defined from hospitalization records in the previous 5 years in the administrative data study, and from records in the patient's chart in the clinical data study) as patient-level variables. Data were analyzed using SAS version 9.3 (SAS Institute Inc.). All tests were two-sided with *p*-values<0.05 denoting statistical significance. Although the administrative data study used the entire population rather than a sample and therefore *p*-values do not apply, the clinical data study was a sample of the population and so for consistency we have included *p*-values for both studies.

### Power and Sample Size

#### Administrative data study

The cohort included in the administrative data study was the entire population aged ≥40 years with diagnosed diabetes in Ontario, which was more than 900,000 people. With this fixed sample size, the study had >95% power to detect an unadjusted absolute difference of at least 0.4% in a dichotomous primary outcome, using an α-error of 0.05. Power would be reduced after adjustment for baseline differences and for clustering, but would remain sufficient to detect even very small differences in outcomes.

#### Clinical data study

The sample size for the clinical data study was calculated a priori on the basis of an ability to detect an absolute 10% difference in statin prescription rates between intervention and control patients, a threshold similar to the median effect size found in a systematic review of printed educational materials [15]. To have 80% power to detect this difference with an α-error of 0.05, a sample size of 796 per group with 20 patients per practice was needed [16].

## Results

**Figure 1:**
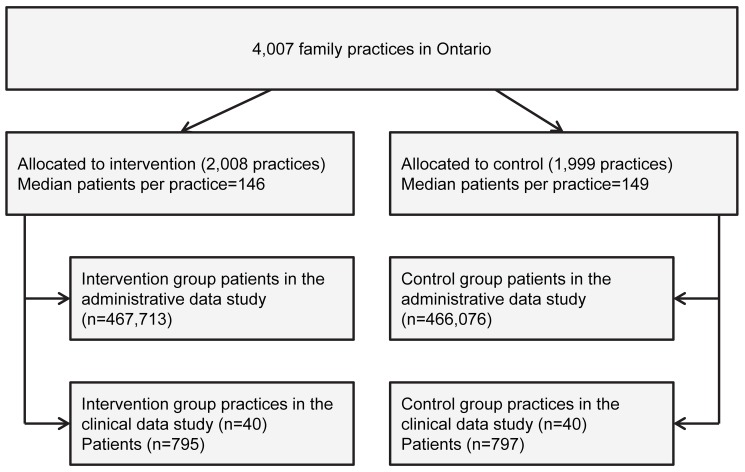
Flow chart of practice and patient randomization in the trial.

We randomized 4,007 family practices in Ontario, to which 933,789 patients with diabetes were assigned in the administrative data study (Figure 1). In the clinical data study, we approached 372 intervention practices and 395 control practices to recruit 40 of each, and we examined the records of 1,592 randomly selected patients at high risk for cardiovascular disease. The pre-specified sample size was met. The baseline characteristics of patients and practices were well balanced (Table 1).

**Table 1:** Patient and practice baseline characteristics, by study group.

Baseline Characteristic	Administrative Data Study		Clinical Data Study	
	Intervention	Control	Intervention	Control
**Patients**				
*n*	467,713	466,076	795	797
Age, mean (SD), y	64.3 (12.4)	64.2 (12.4)	65.9 (10.3)	65.5 (10.6)
Male	246,741 (52.8)	245,204 (52.6)	412 (51.8)	429 (53.8)
Diabetes type^a^				
Type 1			14 (1.8)	11 (1.4)
Type 2			781 (98.2)	786 (98.6)
Diabetes duration, y				
<2	76,547 (16.4)	77,011 (16.5)	145 (18.2)	120 (15.1)
2–<5	112,509 (24.1)	112,543 (24.1)	196 (24.7)	183 (23.0)
5–<10	127,375 (27.2)	126,831 (27.2)	195 (24.5)	214 (26.9)
10+	151,282 (32.3)	149,691 (32.1)	252 (31.7)	275 (34.5)
Previous cardiovascular disease	30,108 (6.4)	29,801 (6.4)	317 (39.9)	331 (41.5)
Hypertension	318,015 (68.0)	317,941 (68.2)	754 (94.8)	767 (96.2)
Baseline CAD assessment^b^				
Electrocardiogram	184,804 (39.5)	190,041 (40.8)	—	—
Cardiac stress test or nuclear imaging	38,540 (8.2)	40,269 (8.6)	—	—
Coronary angiography	8,222 (1.8)	8,060 (1.7)	—	—
Cardiology or internal medicine visit	96,733 (20.7)	97,982 (21.0)	—	—
Baseline medication utilization^b^^,^ c				
ACEI/ARB	152,659 (72.3)	151,629 (72.8)	—	—
≥1 antihypertensive class	179,312 (84.9)	177,153 (85.1)	—	—
≥2 antihypertensive classes	132,008 (62.5)	131,134 (63.0)	—	—
≥3 antihypertensive classes	67,574 (32.0)	68,015 (32.7)	—	—
Statin	145,746 (69.0)	144,395 (69.3)	—	—
Glucose-lowering drug	129,572 (61.4)	127,239 (61.1)	—	—
Insulin	25,826 (12.2)	24,664 (11.8)	—	—
Nitrate	23,187 (11.0)	22,976 (11.0)	—	—
**Practices**				
*n*	2,008	1,999	40	40
Practice type				
Solo	1,125 (56.0)	1,155 (57.8)	16 (40.0)	22 (55.0)
Group	883 (44.0)	844 (42.2)	24 (60.0)	18 (45.0)
Rural practice	190 (9.5)	160 (8.0)	2 (5.0)	1 (2.5)
Diabetes patient volume				
<100	760 (37.8)	708 (35.4)	7 (17.5)	4 (10.0)
100–<200	742 (37.0)	788 (39.4)	23 (57.5)	15 (37.5)
200+	506 (25.2)	503 (25.2)	10 (25.0)	21 (52.5)

**Number (%) except where indicated.**

a Only available in the clinical data study.

b Only available in the administrative data study. Measured in the 10 months prior to the start of follow-up.

c Among patients aged ≥65 years: 211,137 in the intervention group, 208,286 in the control group.

ACEI, angiotensin converting enzyme inhibitor; ARB, angiotensin receptor blocker; CAD, coronary artery disease; SD, standard deviation.

### Administrative Data Study

The primary outcome, death and non-fatal myocardial infarction, occurred in 11,736 (2.5%) patients in the intervention group, and 11,536 (2.5%) patients in the control group (odds ratio 1.00, 95% confidence interval 0.96–1.03, *p* = 0.77). The other clinical event outcomes did not show benefit with the intervention (Table 2). Two of the processes of care secondary outcomes related to coronary artery disease screening were statistically significantly worse in the intervention group: electrocardiograms (38.8% versus 40.2%, odds ratio 0.96, 95% confidence interval 0.93–0.99, *p* = 0.02) and cardiac stress tests or nuclear imaging (7.8% versus 8.1%, odds ratio 0.96, 95% confidence interval 0.93–1.00, *p* = 0.04). The intervention did not significantly increase initiation of medications targeting cardiovascular risk factors or glycemia. Fewer patients in the intervention group were started on nitrates than in the control group (odds ratio 0.96, 95% confidence interval 0.92–1.00, *p* = 0.03).

**Table 2:** Primary and secondary outcomes of the toolkit intervention.

Outcome Measure	Intervention	Control	OR (95% CI)^a^	*p*-Value	ICC
**Administrative data study**					
***Primary outcome***					
Death or non-fatal myocardial infarction	11,736/467,713 (2.5%)	11,536/466,076 (2.5%)	1.00 (0.96–1.03)	0.77	0.003
***Secondary outcomes – clinical events***					
All-cause death	8,704/467,713 (1.9%)	8,704/466,076 (1.9%)	0.98 (0.94–1.01)	0.21	0.002
Myocardial infarction	3,944/467,713 (0.8%)	3,767/466,076 (0.8%)	1.03 (0.97–1.08)	0.34	0.001
Myocardial infarction or unstable angina	5,002/467,713 (1.1%)	4,756/466,076 (1.0%)	1.04 (0.99–1.09)	0.15	0.001
Stroke	1,863/467,713 (0.4%)	1,884/466,076 (0.4%)	0.98 (0.91–1.04)	0.45	<0.001
Stroke or transient ischemic attack	2,254/467,713 (0.5%)	2,273/466,076 (0.5%)	0.98 (0.92–1.04)	0.46	<0.001
Death, non-fatal myocardial infarction, non-fatal stroke	12,981/467,713 (2.8%)	12,773/466,076 (2.7%)	1.00 (0.97–1.03)	0.77	0.003
Death, non-fatal myocardial infarction, non-fatal stroke, unstable angina, or transient ischemic attack	14,330/467,713 (3.1%)	14,051/466,076 (3.0%)	1.00 (0.96–1.04)	0.96	0.004
***Secondary outcomes – CAD assessment***					
Electrocardiogram	181,404/467,713 (38.8%)	187,391/466,076 (40.2%)	0.96 (0.93–0.99)	0.02	0.053
Cardiac stress test or nuclear imaging	36,373/467,713 (7.8%)	37,918/466,076 (8.1%)	0.96 (0.93–1.00)	0.04	0.015
Coronary angiography	7,633/467,713 (1.6%)	7,450/466,076 (1.6%)	1.00 (0.96–1.05)	0.83	0.002
Coronary revascularization procedure	3,540/467,713 (0.8%)	3513/466,076 (0.8%)	0.99 (0394–1.05)	0.81	0.001
Cardiology or internal medicine visit	97,193/467,713 (20.8%)	98,944/466,076 (21.2%)	0.97 (0.94–1.00)	0.07	0.029
***Secondary outcomes – medication initiation*** b					
ACEI/ARB	6,462/58,478 (11.1%)	6,843/56,657 (11.2%)	0.99 (0.95–1.03)	0.65	0.008
≥1 antihypertensive class	4,451/31,825 (14.0%)	4,403/31,133 (14.1%)	0.99 (0.94–1.04)	0.64	0.007
≥2 antihypertensive classes	7,712/79,129 (9.7%)	7,463/77,152 (9.7%)	1.01 (0.97–1.05)	0.59	0.003
≥3 antihypertensive classes	8,377/143,563 (5.8%)	8,176/140,271 (5.8%)	1.00 (0.96–1.04)	0.94	0.002
Statin	8,091/65,391 (12.4%)	7,967/63,891 (12.5%)	1.00 (0.96–1.04)	0.94	0.013
Glucose-lowering drug	6,261/81,565 (7.7%)	6,123/81,047 (7.6%)	1.02 (0.98–1.07)	0.37	0.008
Insulin	4,085/185,311 (2.2%)	3,945/183,622 (2.1%)	1.02 (0.97–1.08)	0.44	0.003
Nitrate	8,726/187,950 (4.6%)	8,936/185,310 (4.8%)	0.96 (0.92–1.00)	0.03	0.003
**Clinical data study**					
***Primary outcome***					
Prescription for statin	700/795 (88.1%)	725/797 (91.0%)	0.73 (0.42–1.26)	0.26	0.123
***Secondary outcomes – cardiovascular risk reduction***					
Prescription for ACEI/ARB	671/795 (84.4%)	689/797 (86.4%)	0.77 (0.51–1.15)	0.20	0.060
HbA1c≤7.0%	465/795 (58.5%)	469/797 (58.8%)	0.93 (0.71–1.21)	0.58	0.038
Blood pressure ≤130/80	420/795 (52.8%)	506/797 (63.5%)	0.72 (0.53–0.98)	0.04	0.089
LDL-cholesterol ≤2.0 mmol/l	471/795 (59.2%)	492/797 (61.7%)	0.90 (0.68–1.18)	0.43	0.040
Total- to HDL-cholesterol ratio ≤4.0	590/795 (74.2%)	612/797 (76.8%)	0.85 (0.63–1.14)	0.27	0.022
***Secondary outcomes – clinical inertia***					
When HbA1c>8.0%	20/170 (11.8%)	25/192 (13.0%)	0.98 (0.48–1.98)	0.95	0.046
When blood pressure >140/90	21/337 (5.6%)	27/371 (7.2%)	0.67 (0.25–1.82)	0.43	0.194
When LDL-cholesterol >3.0 mmol/l	54/124 (43.5%)	52/115 (45.2%)	0.94 (0.53–1.67)	0.83	0.087

a Adjusting for age, sex, diabetes duration, previous cardiovascular disease, and practice diabetes patient volume.

b Among patients aged ≥65 years who were not already receiving the medication at baseline.

ACEI, angiotensin converting enzyme inhibitor; ARB, angiotensin receptor blocker; CAD, coronary artery disease; ICC, intraclass correlation coefficient; OR, odds ratio.

### Clinical Data Study

Seven hundred (88.1%) patients in the intervention group and 725 (90.1%) patients in the control group used a statin (odds ratio 0.73, 95% confidence interval 0.42–1.26, *p* = 0.26). Likewise, there was no difference between groups in the proportion of patients receiving an angiotensin converting enzyme inhibitor or angiotensin receptor blocker (Table 2). Fewer patients in the intervention group reached blood pressure control targets (52.8% versus 63.5%, odds ratio 0.72, 95% confidence interval 0.53–0.98, *p* = 0.04). There was no difference in the proportion reaching glycemic and LDL-cholesterol control targets. Clinical inertia also did not differ between groups. An exploratory analysis also adjusting for practice type (Table S1) gave virtually identical results.

## Discussion

Printed educational materials in the form of a cardiovascular disease toolkit did not improve quality of care or outcomes in a population with diabetes. Some of the secondary outcomes were statistically significantly worse in the intervention group, although we cannot exclude that these represent chance findings due to multiple hypotheses testing. Thus, this quality improvement intervention not only failed to improve care, but if anything may have adversely affected some of the secondary outcomes.

The study has several strengths to highlight. It used a pragmatic randomized trial design to obtain a valid estimate of the effectiveness of the toolkit in real world clinical care, not just in an idealized efficacy trial setting. By randomizing in clusters at the practice level, the risk for contamination was minimized. The study included nearly 1 million patients—the entire population of patients with diabetes in Canada's most populous province—making it the largest ever randomized trial in diabetes. Using administrative data sources to evaluate outcomes ensured complete data collection with no loss to follow-up or missing data. The lack of detailed clinical data available in these data sources was supplemented with data collected directly from patient charts in the clinical data study. However, there are some possible limitations to note. First, the toolkit was developed without a specific quality improvement or educational theory to guide its content or delivery, which might otherwise have increased the likelihood of it leading to clinically important improvements in care [20]. Although the toolkit addressed clinician knowledge, other barriers to implementation of evidence-based care (such as time, patient adherence or comorbidity, or organization of health care delivery) were not addressed [21]. Second, the follow-up time of the study was short, which may have been insufficient time in which to see changes in clinical events. However, process measures and drug prescriptions could very easily be influenced within the short time frame of the study, and yet the intervention had no impact on these secondary outcomes as well. Third, although practices participating in the clinical data study were randomly selected, they may have been subject to volunteer bias as those who agreed to participate could be different from the large number who did not. However, it is unlikely that this would influence the validity of the study, as such bias would have affected both intervention and control practices equally. Finally, in the clinical data study, utilization of statins and of angiotensin converting enzyme inhibitors or angiotensin receptor blockers were both >85%, even in the control group. Thus, utilization may already have reached a ceiling, beyond which further improvements from an intervention would be unlikely.

The possibility that the intervention might have worsened care is unexpected. It is of course possible that the four statistically significant findings showing harm were chance findings due to multiple hypothesis testing, but notably there were no results in the opposite direction. Therefore, we feel it necessary to speculate on possible explanations for why this intervention could have worsened care, should these findings be real. Although its content was focused on cardiovascular disease, the toolkit was disseminated by the Canadian Diabetes Association and it was temporally linked with the newly released national diabetes practice guidelines. Hence, the intervention may have inadvertently focused the attention of physicians onto other aspects of diabetes care, and away from broad cardiovascular disease risk management, which might have been perceived of as a “mere” comorbidity of diabetes. Lack of attention to cardiovascular risk factor management would be detrimental to patient outcomes, especially since cardiovascular disease is the most common cause of death for diabetic patients [2],[22],[23]. These findings highlight the importance of monitoring for unintended consequences of quality improvement interventions [24].

The previous literature has demonstrated that the benefits of printed educational interventions are, at best, modest. A systematic review of methods to improve practice guideline adherence demonstrated an absolute improvement of 8% for educational materials [15]. A more recent Cochrane review found that printed educational materials led to a median absolute improvement in performance of only 2% [25]. A diabetes-specific review of quality improvement strategies to improve glycemic control showed that clinician education interventions had the lowest impact of any quality improvement approach, with a mean reduction in HbA1c of 0.16% [26]. Studies of printed materials specifically tied to clinical practice guidelines also showed modest benefits. A small English study randomized 42 family physicians to receive an algorithm for monitoring and treatment of hypertension of diabetic patients based on practice guidelines, but found no difference in blood pressure control between the intervention and control groups [27]. However, some processes of care were slightly improved: patients in the intervention group were prescribed higher doses of antihypertensive medications, and had more physician visits to monitor blood pressure. In a larger Canadian study, family physicians were randomized to receive a one-page summary of a three-year-old practice guideline on anti-anginal therapy from the local medical governing body [28]. No differences were noted in prescription of β-blockers, antiplatelet agents, or lipid-lowering drugs between groups in the 7,000 patients reviewed. We had hoped that the intervention in our study might have had greater impact because it was produced and disseminated by the CDA, an advocacy and professional organization with credibility among family physicians for diabetes messaging, and because the intervention was released in conjunction with a well-publicized update to national diabetes practice guidelines, increasing the salience of the intervention and its content. However, these differences of this intervention from those previously studied did not result in improved outcomes.

In summary, the cardiovascular disease management toolkit failed to reduce clinical events or improve quality of care for patients with diabetes. It may actually have led to worsening in some secondary outcomes, though this may represent a chance finding. Despite years of evidence that printed educational materials have, at best, only a modest impact on quality of care, they remain a very commonly used intervention for quality improvement. Therefore, even the remote potential for them to cause true harm is worrying, as the public health consequences could be significant. Quality improvement interventions are often developed by clinicians or policy makers in an unsystematic way. The results of this study highlight the need for a rigorous and scientifically based approach to the development, dissemination, and evaluation of quality improvement interventions.

## Supporting Information

Table S1 **Results of an exploratory analysis of the clinical data study, with additional adjustment for practice type.**
(DOCX)

Text S1 **Trial protocol for the clinical data study.**
(PDF)

Text S2 **Trial protocol for the administrative data study.**
(DOC)

Text S3 **CONSORT statement.**
(DOCX)

## Abbreviations

CDA: Canadian Diabetes Association
ODD: Ontario Diabetes Database
