# Supplementary material for: Effect of an Educational Toolkit on Quality of Care: A Pragmatic Cluster Randomized Trial
Source: PLoS Med. 2014 Feb 4;11(2):e1001588. doi: 10.1371/journal.pmed.1001588 (PMC3913553; doi:10.1371/journal.pmed.1001588)
Supplement: Table S1 — Results of an exploratory analysis of the clinical data study, with additional adjustment for practice type. (DOCX) [file pmed.1001588.s001.docx]

| Outcome measure | OR (95% CI)^a^ | *P* value |
| --- | --- | --- |
| *Primary outcome* |  |  |
| Prescription for statin | 0.69 (0.38–1.26) | 0.23 |
| *Secondary outcomes – cardiovascular risk reduction* |  |  |
| Prescription for ACEI / ARB | 0.78 (0.53–1.15) | 0.21 |
| HbA1c ≤7.0% | 0.94 (0.71–1.25) | 0.68 |
| Blood pressure ≤130/80 | 0.67 (0.49–0.91) | 0.01 |
| LDL-cholesterol ≤2.0 mmol/L | 0.88 (0.66–1.16) | 0.36 |
| Total- to HDL-cholesterol ratio ≤4.0 | 0.84 (0.62–1.14) | 0.27 |
| *Secondary outcomes – clinical inertia* |  |  |
| When HbA1c >8.0% | 0.93 (0.45–1.93) | 0.85 |
| When blood pressure >140/90 | 0.67 (0.23–1.96) | 0.46 |
| When LDL-cholesterol >3.0 mmol/L | 0.89 (0.49–1.60) | 0.69 |

^a^ adjusting for age, sex, diabetes duration, previous cardiovascular disease, practice diabetes patient volume and practice type

OR = odds ratio; CI = confidence interval; ACEI = angiotensin converting enzyme inhibitor; ARB = angiotensin receptor blocker
