# Supplementary material for: Effect of an Educational Toolkit on Quality of Care: A Pragmatic Cluster Randomized Trial
Source: PLoS Med. 2014 Feb 4;11(2):e1001588. doi: 10.1371/journal.pmed.1001588 (PMC3913553; doi:10.1371/journal.pmed.1001588)
Supplement: Text S1 — Trial protocol for the clinical data study. (PDF) [file pmed.1001588.s002.pdf]

Shah et al. Trials 2010, 11:44
http://www.trialsjournal.com/content/11/1/44

TRIALS

STUDY PROTOCOL Open Access

Evaluation of a toolkit to improve cardiovascular disease screening and treatment for people with type 2 diabetes: protocol for a cluster-randomized pragmatic trial

Baiju R Shah*[1,2,3], Onil Bhattacharyya[1,4], Catherine Yu[1,4], Muhammad Mamdani[1,2,4], Janet A Parsons[1,4], Sharon E Straus[1,4] and Merrick Zwarenstein[1,2,3]

Abstract

Background: The gap between the level of care recommended by evidence-based clinical practice guidelines and the actual care delivered to patients in practice has been well established. The Canadian Diabetes Association (CDA) created an implementation strategy to improve the implementation of its 2008 guidelines. This study will evaluate the impact of the strategy to improve cardiovascular disease (CVD) screening, prevention and treatment for people with diabetes.

Design: A pragmatic cluster-randomized trial will be conducted to evaluate the CDA's CVD Toolkit. All family physicians in Ontario, Canada were randomly allocated to receive the Toolkit, which includes several printed educational materials targeting CVD screening, prevention and treatment, either in spring 2009 (intervention arm) or in spring 2010 (control arm). Randomization occurred at the level of the practice. Forty family physicians from each arm will be recruited to participate, and the medical records for 20 of their diabetic patients at high risk for CVD will be retrospectively reviewed. Outcome measures will be assessed for each patient between July 2009 and March 2010. The primary outcome will be that the patient is receiving a statin. Secondary outcomes will include 1) the receipt of an angiotensin converting enzyme inhibitor or angiotensin receptor blocker, 2) various intermediate measures (A1c, blood pressure, LDL-cholesterol, total-/HDL-cholesterol ratio, body mass index and waist circumference), and 3) clinical inertia (the failure to change therapy in response to an abnormal A1c, blood pressure or cholesterol reading). The analysis will be carried out using multilevel hierarchical logistic regression models to account for the clustered nature of the data. The group assignment will be a physician-level variable. In addition, a process evaluation study with six focus groups of family physicians will assess the acceptability of the CDA's Toolkit and will explore factors contributing to any change or lack of change in behaviour, from the perspectives of family physicians.

Discussion: Printed educational materials for physicians have been shown to exert small-to-moderate changes in patient care. The CDA's CVD Toolkit is an example of a practice guideline implementation strategy that can be disseminated to a wide audience relatively inexpensively, and so demonstrating its effectiveness at improving diabetes care could have important consequences for guideline developers, policy makers and clinicians.

Trial Registration: The trial is registered with http://www.clinicaltrials.gov, ID # NCT01026688

Background

Diabetes is a common and serious chronic disease. Its prevalence is growing extremely rapidly, [1] and it is associated with impaired quality of life, premature mortality and significant economic costs [2-4]. One of the most important complications of diabetes is cardiovascular disease, as it accounts for about half of mortality in people with diabetes [3]. Fortunately, numerous randomized trials have shown that pharmacological and non-pharma-

* Correspondence: baiju.shah@ices.on.ca
[1] University of Toronto, Toronto, Ontario, Canada
Full list of author information is available at the end of the article

BioMed Central

© 2010 Shah et al; licensee BioMed Central Ltd. This is an Open Access article distributed under the terms of the Creative Commons Attribution License (http://creativecommons.org/licenses/by/2.0), which permits unrestricted use, distribution, and reproduction in any medium, provided the original work is properly cited.

cological approaches can reduce the risk of complications and morbidity for people with diabetes[5-8]. In particular, multi-faceted interventions that target behaviour modification, glycemia and cardiovascular risk factors substantially reduce cardiovascular events and mortality[9,10]. However, these approaches are complex, as they require management of the multiple risk factors that lead to complications. Clinical practice guidelines (such as those produced by the Canadian Diabetes Association [CDA]) can help health care providers and patients by synthesizing the enormous literature on diabetes management into specific recommendations for care. However, many studies have previously documented a gap between these recommendations and the actual care delivered to patients with diabetes in Canada[11-14]. The lack of impact of practice guidelines on actual clinical practice is not unique to diabetes -- studies in a variety of clinical areas have demonstrated this lack of effect[15-17]. Therefore, the implementation of evidence-based guideline recommendations in actual clinical practice needs to be improved.

For the 2008 update to the guidelines, the CDA formed a Dissemination and Implementation Committee to create a guideline implementation strategy[18]. The first component of this strategy was aimed at improving adherence with the recommendations for cardiovascular disease screening and treatment for people with diabetes, since this is the most important complication of the disease and since evidence-based preventive interventions are readily available. The strategy highlighted the identification of diabetic patients at high risk for cardiovascular events, treatment targets and methods for vascular protection, and the selection of patients and methods for coronary artery disease screening. The objective of this study is to evaluate the impact of the CDA's strategy to improve cardiovascular disease screening, prevention and treatment for people with diabetes.

Methods and design

Research design

We will conduct a pragmatic cluster-randomized controlled trial to evaluate the effectiveness of the CDA's physician-oriented cardiovascular disease toolkit to improve the management of cardiovascular risk factors for patients with diabetes. A "pragmatic attitude" to trial design leads to a different type of trial answering different questions than a traditional "explanatory attitude" to design[19,20]. Explanatory randomized controlled trials (RCTs) are conducted in well-resourced, idealized clinical settings with motivated clinicians focusing on the particular clinical problem, whereas pragmatic trials are conducted in real-world clinical care in a variety of practice settings. Patients in explanatory RCTs are very highly selected with stringent inclusion and exclusion criteria, whereas very little selection is done in pragmatic RCTs, leading to study populations that more closely reflect the range of patients seen in clinical practice. Interventions are strictly defined and enforced with close monitoring of adherence in explanatory RCTs, while they are applied more flexibly in pragmatic RCTs. Finally, explanatory RCTs usually have a single primary outcome that is the focus of the study, whereas pragmatic RCTs often examine a broad range of endpoints that may have relevance to patients, clinicians and decision makers. As a result of these differences, explanatory RCTs are useful to establish *efficacy* of treatment, whereas pragmatic RCTs can establish *effectiveness*. Pragmatic trials are thus particularly useful to evaluate strategies and population-based initiatives, such as the knowledge translation strategy being evaluated in this study.

Intervention

The cardiovascular disease toolkit was packaged in a brightly-coloured box with CDA branding. (See Figure 1.) The contents included an introductory letter from the Chair of the practice guidelines' Dissemination and Implementation Committee; an eight page summary of selected sections of the practice guidelines targeted towards primary care physicians; a four page synopsis of the key guideline elements pertaining to cardiovascular disease risk; a small double-sided laminated card with a simplified algorithm for cardiovascular risk assessment, vascular protection strategies and screening for cardiovascular disease; and a pad of tear-off sheets for patients with a cardiovascular risk self-assessment tool and a list of recommended risk reduction strategies. (See Figure 2.)

In the intervention group, the Toolkit was mailed with the Spring 2009 edition of *Canadian Diabetes*, a newsletter from the CDA which provides practical information on diagnosis and treatment issues associated with diabe-

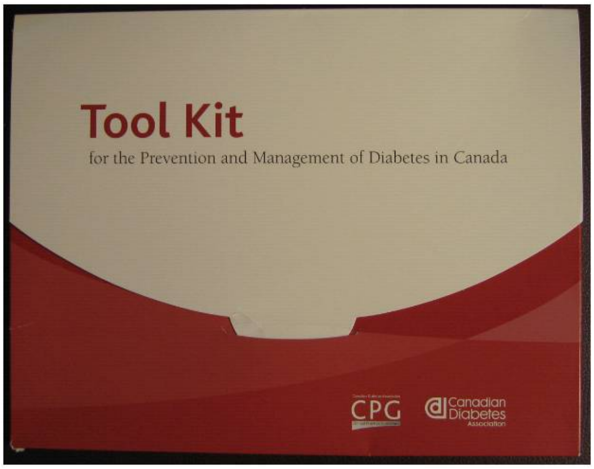

Figure 1 **Packaging for the cardiovascular toolkit intervention**.

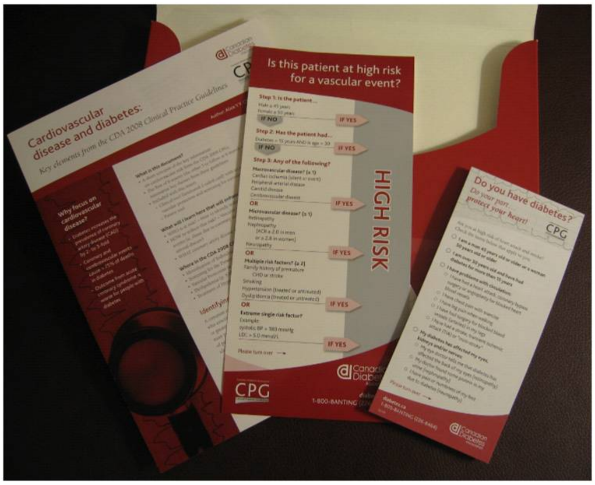

Figure 2 Contents of the Toolkit included (left to right): a synopsis of the guideline elements pertaining to cardiovascular disease risk, a laminated card with a simplified algorithm for cardiovascular risk management, and a pad of tear-off sheets for patients with a cardiovascular risk self-assessment tool.

tes that is sent quarterly to all primary care physicians in Canada. The content of this edition of the newsletter did not pertain to cardiovascular risk screening or treatment. Both the Toolkit and *Canadian Diabetes* were packaged together in a large mailing envelope. The control group received *Canadian Diabetes* alone in its usual shrink-wrap packaging, and will receive the Toolkit with the Spring 2010 edition of the newsletter.

Allocation of participants

All family physicians in Ontario were randomized to either the intervention or control group using computer-generated randomization. Randomization occurred at the level of the practice based on the physicians' primary practice address recorded with the College of Physicians and Surgeons of Ontario, so that all physicians practicing at a single location were assigned to the same group. This was done to prevent contamination between physicians in group practices who may share information and resources. The randomization was stratified by health region.

In addition to the randomized distribution to family physicians in Ontario, the Toolkit was also distributed to all endocrinologists in Ontario, and to all family physicians and endocrinologists in all other Canadian provinces and territories in a non-randomized fashion. Endocrinologists will not directly form part of the study.

Study participants

From the Toolkit's mailing lists, we will randomly select Ontario family physicians to approach about participating in a primary data abstraction study. Physicians will be contacted by fax with a brief synopsis of the study purpose and the planned data abstraction. They will be invited to accept or decline to participate by fax or telephone. Follow-up telephone calls will be made to physicians who do not respond. We will recruit 40 family physicians from each of the intervention and control arms. For practical reasons, the recruited physicians will practice within 150 km of the study centre, which incorporates both urban and rural areas. In order to increase the likelihood that the participating physicians are representative of typical practitioners and are not self-selected towards good performers with an interest in diabetes quality of care, the letter of invitation will not describe the study in detail or what data will be collected.

Individual patients with diabetes seeing each physician will be identified through the physician's billing records or electronic health records. We will randomly select from these records 20 diabetic patients per physician aged = 18 years who were seen in the office at least once between July 2009 and March 2010, and who fulfill the Clinical Practice Guidelines' definition of being at "high risk for CV events": [18]

- Men aged ≥ 45 years, women aged ≥ 50 years; or
- Men aged < 45 years and women < 50 years with at least one of the following:
  - Macrovascular disease (silent myocardial infarction, or evidence of peripheral arterial, carotid arterial or cerebrovascular disease)
  - Microvascular disease (nephropathy or retinopathy)
  - Family history of premature coronary or cerebrovascular disease in a first-degree relative
  - Extreme level of a single risk factor (LDL-C > 5.0 mmol/L, systolic BP > 180 mmHg)
  - Duration of diabetes > 15 years with age > 30 years

Patients who were concomitantly seeing endocrinologists will not be specifically excluded from the study. Although such patients may be more likely to have received benefit from the Toolkit since all endocrinologists received the Toolkit in a non-randomized fashion, less than 18% of people with diabetes in Ontario see both endocrinologists and family physicians[21]. Any such patients would bias the results of the study towards the null.

Data collection and outcomes

Starting in the spring of 2010, we will perform a retrospective data abstraction from the paper charts and/or electronic health records in each physician's office. For each patient, demographic information (including date of birth, sex and postal code of home residence) and clinical information (including diabetes duration, comorbidities, family history and baseline drug utilization) will be collected. Outcome measures will be assessed for each

patient for the period between July 2009 and March 2010. The primary outcome will be that the patient is receiving a statin (as determined by the record of a new prescription, the record of a prescription renewal, or a notation that the patient is taking a statin). This outcome is selected because as a process of care outcome, it is closely linked to physicians' behaviour and hence is more likely to change as a result of the intervention, and because no more than one-third of patients currently receive statins, [13,22,23] so there is substantial opportunity for improvement.

The other process outcome that will be evaluated is whether the patient is receiving a prescription for an angiotensin-converting enzyme inhibitor or angiotensin receptor blocker. In addition, the following intermediate outcomes will also be collected:

- A1c
- Blood pressure
- LDL-cholesterol
- Total-/HDL-cholesterol ratio
- Body mass index
- Waist circumference

When there are multiple measurements for these intermediate measures for a patient, the last one during the observation period will be used.

We will also measure "clinical inertia"[24]. For each A1c, blood pressure or LDL-cholesterol reading that is above the recommended clinical practice guideline target, we will determine whether, at the next patient visit, there is notation in the chart of a recommended change: the addition of a new medication, a dose change for an existing medication, a dietary or physical activity recommendation, etc.

The data collection will be conducted using a laptop computer by a trained and experienced registered nurse, blinded to group status. To protect confidentiality of personal health information, all information collected through the data abstraction will be protected with software that encrypts the data as they are entered. No identifying information will be collected; instead, unique study numbers will be used for each individual. Separately, each study subject's unique health card number will be collected to permit future linkage with secondary data sources. All data will be uploaded weekly via a secure Internet connection to the study centre.

Proposed sample size

We will power the study to be able to detect an absolute 10% difference in statin prescription rates between groups, a threshold similar to the median effect size found in a systematic review of printed educational materials[25]. Even this modest improvement would be an important achievement at a population level. To have 80% power to detect this difference with alpha-error of 0.05, a sample size of 408 per group would be needed. However, this sample size must be inflated by a variance inflation factor (VIF) to ensure adequate power when taking clustering into account using generalized estimating equation models[26]. The VIF is given by VIF = $1 + (n\text{-}1) \times \rho$, where n is the mean cluster size and ρ is the intraclass correlation coefficient, a measure of the degree of correlation of the outcome within clusters. Previous data have shown that ρ for appropriate statin use by diabetic patients clustered within primary care physicians is 0.05[27]. If we assume a cluster size of 20 eligible patients per physician (which represents a reasonable number of patients whose data could be abstracted in two days), the VIF is $1 + 0.05 \times (20\text{-}1) = 1.95$, so the actual required sample size is 796 patients in each group, or 40 physicians per group.

Planned analyses

We hypothesize that patients whose family physicians are in the intervention arm will have greater use of appropriate medications and better achievement of targets than patients whose family physicians are in the control arm. The analysis to test this hypothesis will be carried out using multilevel hierarchical logistic regression models to account for the clustered nature of the data, i.e., patients clustering within physicians and the attendant reduction in statistical independence. The group assignment will be a physician-level variable.

Process evaluation study

To facilitate the interpretation and generalizability of the quantitative evaluations of the Toolkit's effectiveness, additional information will be sought about the processes and causal mechanisms through which they led or did not lead to physician behaviour change. In a qualitative study to follow the randomized trial, we will first assess the acceptability of the cardiovascular tools (i.e., the usefulness of the tools from physicians' perspectives). Second, we will explore the processes underlying any change or lack of change in behaviour from the perspectives of family physicians. It is possible that there are multiple factors influencing the uptake of the educational tools, physicians' understanding of them, as well as their ability and interest in changing practice. For example, characteristics of physician-patient relationships, features of their practice environments and that of the broader health care system, or their understanding of the clinical problem itself could all be influencing practice behaviour and the feasibility of practice change. Qualitative methods are well-suited to understanding the lived experiences of practitioners in context and will be employed in the process evaluation study [28,29].

Six focus groups will be conducted, each consisting of 8 to 10 family physicians, to evaluate the acceptability,

usability, sustainability, strengths and weaknesses of the CDA's Toolkit. Focus group participants will be selected using purposive sampling in order to obtain the perspectives of those with a range of practice experiences (principle of sample heterogeneity, in keeping with qualitative methodology)[30,31]. Participants with varying practice types (group versus solo), years in practice, and whether or not the physician self-identifies as a user of the Toolkit will be sought[32,33]. A total sample size of six focus groups (n = 48 to 60 participants) is likely to be sufficient to achieve theoretical saturation[34].

Focus groups will be conducted in various locales, in order to determine the extent to which practice setting influences the uptake of the educational tools. While the majority of focus groups will be conducted in Toronto and the surrounding suburban area for reasons of feasibility and economy, one focus group will be conducted in a different metropolitan area and a second will be conducted in a more rural/non-urban locale. Participants will be asked for their perspectives on cardiovascular disease risk assessment and management as currently practiced and barriers/facilitators to the uptake of the educational tools. The Toolkit will then be presented with a simulated case, and participants will discuss the case as a group, working together on a task that simulates real clinical usage. Groups will discuss the characteristics of the Toolkit that lead to their acceptability (or lack thereof), and will discuss what elements would be useful in future CDA Toolkits. The influence of pharmaceutical company sponsorship of the Toolkit will be discussed. Using the Theory of Planned Behaviour as a basis, the focus groups will discuss the three variables known empirically to predict behaviour: attitude (being in favour of or against doing something), subjective norms (perceived pressure from social sources to do or not to do something), and perceived behavioural control (perception of having or not having control over their behaviour)[35].

Focus groups will be based on interview guides developed by experienced qualitative researchers, using the trial results as a basis for development. Face and content validity of the guides will be assessed by other team members via pilot testing. Experienced qualitative researchers will conduct the focus groups, which is essential to manage group dynamics, minimize social desirability bias, and probe emergent findings *in situ*. All interviews will be audio taped and transcribed verbatim and detailed field notes will be kept of all interviews, providing two robust sources of data for analysis. Written transcripts will be analyzed for emergent categories and themes using constant comparison within and across interviews[36]. Data management will be facilitated using NVivo software (version 8).

Techniques for ensuring analytic rigour will entail questioning, checking and theorizing in the manner outlined by Kvale[36]. Multiple readings of the transcripts, with subsets of transcripts reviewed by different members of the qualitative research team and an independent researcher to interrogate the developing coding scheme and emergent analysis will be employed[37]. Alternative explanations of the data will be explored in order to develop the most plausible and robust interpretation of the interview findings[37]. Finally, the qualitative data will be compared with the quantitative datasets in an effort to triangulate and understand these results within the context of the study as a whole[38].

Research ethics

The study has been approved by the Research Ethics Board of Sunnybrook Health Sciences Centre, reference number 421-2009.

Discussion

Gaps between the level of diabetes care recommended by evidence-based practice guidelines and the actual care delivered in clinical settings are well known. Studies using large American national health surveys have shown that, at a population level, diabetic patients have poor control of vascular risk factors[39]. Only one-third of patients had A1c levels on target (<7.0%), while more than one in five had poor control (>9.0%). Similarly, only about one-third achieved target blood pressure (<130/80) while 40% had poor control (>140/90), and half had LDL-cholesterol levels above target. Similarly poor management of cardiovascular risk factors has been found in population-level studies of diabetic patients in other countries [13,40-43]. Therefore, strategies to improve the uptake of evidence-based clinical practice guidelines for diabetes and to increase the number of patients meeting treatment targets are urgently needed to reduce the cardiovascular risk for these patients.

The Toolkit to be evaluated in this study is a form of printed educational materials. The effect of printed educational materials to improve diabetes care is uncertain. A meta-regression analysis by Shojania et al. [44] examined several different quality improvement interventions to determine their relative effectiveness to improve glycemic control among people with type 2 diabetes. Most types of interventions led to small-to-moderate improvements in glycemic control. Clinician education interventions, which included printed educational materials, led to a reduction in A1c of between 0.4 and 0.5%. However, once the effect of other simultaneous quality improvement interventions was taken into account, the effect of clinician education on A1c was attenuated (reduction by 0.15%), and was no longer statistically significant. In clinical areas outside of diabetes, the effectiveness of printed

educational materials for clinicians to improve evidence-based care and guideline adherence has been reviewed. Several recent reviews have shown that they can exert a small to moderate influence to improve care[25,45,46] Grimshaw et al. [25] reviewed 235 studies examining guideline dissemination and implementation strategies published between 1976 and 1998. They found five comparisons of educational materials as a single intervention that reported dichotomous process data, and the median effect was an 8.1% absolute improvement in performance (range 3.6% to 17%). A more recent Cochrane review, that included studies published until 2006, found 23 studies that compared printed educational material as a single intervention versus no intervention[46]. The reviewed randomized trials led to a median absolute improvement in performance of 4.3% for categorical process measures and a relative improvement of 13.6% for continuous process measures.

Thus, previous research has generally supported that clinician education via printed educational materials may lead to modest improvements in quality of care. However, all reviews highlighted some fundamental problems with the primary evidence: the likelihood of publication bias, and the fact that the reviews' results were drawn from a small number of studies, most of which were underpowered and had important methodological weaknesses, such as inherent flaws in the study design or unit of analysis errors. Indeed, Grimshaw commented: "We would not conclude that printed educational materials are effective, given the methodologic weaknesses of the primary studies." [25] Instead, they stated that educational materials *may* be effective, and given their relatively low cost and feasibility, they should be considered to improve care. Therefore, rigorous studies are needed to examine the true effectiveness of printed educational materials to drive changes in practice. Large trials of printed educational materials in this population are ongoing, [47], but there are several reasons why this trial may be expected to yield different results. First, the source of the interventions in our study is the CDA, an advocacy and professional organization that would be reputable for diabetes messaging among primary care physicians. Second, educational materials accompanying the release of practice guidelines with significant publicity might have a greater impact than educational materials released in isolation, as the clinical problem and the physician community's response would be more salient now. Third, one part of the Toolkit is a practical and directive decision-making tool with a straightforward algorithm, which may be more actionable for clinicians than materials simply urging behaviour change. Hence, an evaluation of the CDA's Toolkit is an important contribution to the knowledge translation literature, because of the source, timing and content of the materials.

Since guidelines on their own are not beneficial, effective implementation strategies for the target audience should accompany their development. Understanding the effectiveness of this particular approach will be useful for guideline developers and government organizations when limited funds are available to reach large numbers of people. The study will increase understanding of whether this level of intensity of implementation strategy and this combination of factors in an intervention can change behaviour, which will guide future implementation research.

Abbreviations
CDA: Canadian Diabetes Association; RCT: Randomized controlled trial; VIF: Variance inflation factor.

Competing interests
The authors declare that they have no competing interests.

Authors' contributions
BRS conceived of the study and drafted the manuscript. All authors participated in the design of the study, revised the manuscript critically for intellectual content, and have read and approved the final manuscript.

Acknowledgements
The study will be funded by the Canadian Institutes of Health Research (CIHR). Dr. Shah is supported by the CIHR, the Canadian Diabetes Association and the Banting and Best Diabetes Centre, University of Toronto.

Author Details
[1]University of Toronto, Toronto, Ontario, Canada, [2]Institute for Clinical Evaluative Sciences, Toronto, Ontario, Canada, [3]Sunnybrook Research Institute, Sunnybrook Health Sciences Centre, Toronto, Ontario, Canada and [4]Li Ka Shing Knowledge Institute, St. Michael's Hospital, Toronto, Ontario, Canada

Received: 11 December 2009 Accepted: 23 April 2010
Published: 23 April 2010

References
1. Lipscombe LL, Hux JE: **Trends in diabetes prevalence, incidence, and mortality in Ontario, Canada 1995-2005: a population-based study.** *The Lancet* 2007, **369:**750-756.
2. Stewart AL, Greenfield S, Hays RD, Wells K, Rogers WH, Berry SD, McGlynn EA, Ware JE Jr: **Functional status and well-being of patients with chronic conditions. Results from the Medical Outcomes Study.** *JAMA: Journal of the American Medical Association* 1989, **262:**907-913.
3. Gregg EW, Gu Q, Cheng YJ, Narayan KMV, Cowie CC: **Mortality trends in men and women with diabetes, 1971 to 2000.** *Ann Intern Med* 2007, **147:**149-155.
4. Dawson KG, Gomes D, Gerstein H, Blanchard JF, Kahler KH: **The economic cost of diabetes in Canada, 1998.** *Diabetes Care* 2002, **25:**1303-1307.
5. Diabetes Control and Complications Trial Research Group: **The effect of intensive treatment of diabetes on the development and progression of long-term complications in insulin-dependent diabetes mellitus.** *The New England Journal of Medicine* 1993, **329:**977-986.
6. UK Prospective Diabetes Study Group: **Tight blood pressure control and risk of macrovascular and microvascular complications in type 2 diabetes (UKPDS 38).** *BMJ* 1998, **317:**703-713.
7. UK Prospective Diabetes Study Group: **Intensive blood-glucose control with sulphonylureas or insulin compared with conventional treatment and risk of complications in patients with type 2 diabetes (UKPDS 33).** *The Lancet* 1998, **352:**837-853.
8. The ADVANCE Collaborative Group: **Intensive blood glucose control and vascular outcomes in patients with type 2 diabetes.** *The New England Journal of Medicine* 2008, **358:**2560-2572.
9. Gæde P, Vedel P, Larsen N, Jensen GVH, Parving HH, Pedersen O: **Multifactorial intervention and cardiovascular disease in patients with type 2 diabetes.** *The New England Journal of Medicine* 2003, **348:**383-393.

10. Gæde P, Lund-Andersen H, Parving HH, Pedersen O: **Effect of a multifactorial intervention on mortality in type 2 diabetes.** *The New England Journal of Medicine* 2008, **358:**580-591.
11. Harris SB, Stewart M, Brown JB, Wetmore S, Faulds C, Webster-Bogaert S, Porter S: **Type 2 diabetes in family practice: room for improvement.** *Can Fam Physician* 2003, **49:**778-785.
12. Harris SB, Ekoé J-M, Zdanowicz Y, Webster-Bogaert S: **Glycemic control and morbidity in the Canadian primary care setting (results of the Diabetes in Canada Evaluation study).** *Diabetes Res Clin Pract* 2005, **70:**90-97.
13. Chan BTB, Klomp H, Cascagnette P: *Quality of diabetes management in Saskatchewan* Saskatoon: Health Quality Council; 2006.
14. Shah BR, Hux JE, Laupacis A, Zinman B, Zwarenstein M: **Deficiencies in the quality of diabetes care: comparing specialist with generalist care misses the point.** *J Gen Intern Med* 2007, **22:**275-279.
15. Latosinsky S, Fradette K, Lix L, Hildebrand K, Turner D: **Canadian breast cancer guidelines: Have they made a difference?** *CMAJ* 2007, **176:**771-776.
16. Tu K, Mamdani MM, Tu JV: **Hypertension guidelines in elderly patients: is anybody listening?** *Am J Med* 2002, **113:**52-58.
17. Vernacchio L, Vezina RM, Mitchell AA: **Management of acute otitis media by primary care physicians: trends since the release of the 2004 American Academy of Pediatrics/American Academy of Family Physicians Clinical Practice Guideline.** *Pediatrics* 2007, **120:**281-287.
18. Canadian Diabetes Association Clinical Practice Guidelines Expert Committee: **Canadian Diabetes Association 2008 clinical practice guidelines for the prevention and management of diabetes in Canada.** *Can J Diabetes* 2008, **32:**S1-S201.
19. Zwarenstein M, Treweek S, Gagnier JJ, Altman DG, Tunis S, Haynes B, Oxman AD, Moher D, for the CONSORT and Pragmatic Trials in Healthcare (Practihc) groups: **Improving the reporting of pragmatic trials: an extension of the CONSORT statement.** *BMJ* 2008, **337:**a2390.
20. Tunis SR, Stryer DB, Clancy CM: **Practical clinical trials: increasing the value of clinical research for decision making in clinical and health policy.** *JAMA: Journal of the American Medical Association* 2003, **290:**1624-1632.
21. Jaakkimainen L, Shah BR, Kopp A: **Sources of physician care for people with diabetes.** In *Diabetes in Ontario: An ICES Practice Atlas* Edited by: Hux JE, Booth GL, Slaughter PM, Laupacis A. Toronto: Institute for Clinical Evaluative Sciences; 2003:9.181-9.192.
22. Shah BR, Mamdani M, Kopp A: **Drug use in older people with diabetes.** In *Diabetes in Ontario: An ICES Practice Atlas* Edited by: Hux JE, Booth GL, Slaughter PM, Laupacis A. Toronto: Institute for Clinical Evaluative Sciences; 2003:3.51-3.76.
23. Sirois C, Moisan J, Poirier P, Grégoire JP: **Suboptimal use of cardioprotective drugs in newly treated elderly individuals with type 2 diabetes.** *Diabetes Care* 2007, **30:**1880-1882.
24. Shah BR, Hux JE, Laupacis A, Zinman B, van Walraven C: **Clinical inertia in response to inadequate glycemic control: Do specialists differ from primary care physicians?** *Diabetes Care* 2005, **28:**600-606.
25. Grimshaw J, Eccles M, Thomas R, MacLennan G, Ramsay C, Fraser C, Vale L: **Towards evidence-based quality improvement: evidence (and its limitation) of the effectiveness of guideline dissemination and implementation strategies 1966-1998.** *J Gen Intern Med* 2006, **21:**S14-S20.
26. Donner A, Klar N: *Design and Analysis of Cluster Randomization Trials in Health Research* New York: Oxford University Press; 2000.
27. Krein SL, Hofer TP, Kerr EA, Hayward RA: **Whom should we profile? Examining diabetes care practice variation among primary care providers, provider groups, and health care facilities.** *Health Serv Res* 2002, **37:**1159-1180.
28. Kuper A, Reeves S, Levinson W: **An introduction to reading and appraising qualitative research.** *BMJ* 2008, **337:**404-407.
29. Pope C, Mays N: **Qualitative research: Reaching the parts other methods cannot reach: an introduction to qualitative methods in health and health services research.** *BMJ* 1995, **311:**42-45.
30. Charmaz K: **Grounded theory: Objectivist and constructivist methods.** Edited by: Denzin NK, Lincoln YS. Thousand Oaks: Sage Publications Inc; 2000:509-535.
31. Robson C: *Real World Research: A Resource for Social Scientists and Practitioner-Researchers* Oxford: Blackwell Publishers Ltd; 1993.
32. Madriz E: **Focus groups in feminist research.** In *Handbook of Qualitative Research* Edited by: Denzin NK, Lincoln YS. Thousand Oaks: Sage Publications, Inc; 2000:835-850.
33. **Health Communication Unit. Using Focus Groups Workbook, Version 2.0.** Toronto, Centre for Health Promotion, University of Toronto; 2002.
34. Ryan GW, Bernard HR: **Data management and analysis methods.** Edited by: Denzin NK, Lincoln YS. Thousand Oaks: Sage Publications Inc; 2000:769-802.
35. Ajzen I: **The theory of planned behavior.** *Organ Behav Hum Decis Process* 1991, **50:**179-211.
36. Kvale S: *InterViews: an introduction to qualitative research interviewing* Thousand Oaks, CA: Sage Publications; 1996.
37. Eakin JM, Mykhalovskiy E: **Reframing the evaluation of qualitative health research: reflections on a review of appraisal guidelines in the health sciences.** *J Eval Clin Pract* 2003, **9:**187-194.
38. Bryman A: *Quantity and Quality in Social Research* London: Routledge; 1988.
39. Saydah SH, Fradkin J, Cowie CC: **Poor control of risk factors for vascular disease among adults with previously diagnosed diabetes.** *JAMA: Journal of the American Medical Association* 2004, **291:**335-342.
40. Vaccaro O, Boemi M, Cavalot F, De Feo P, Miccoli R, Patti L, Rivellese AA, Trovati M, Ardigò D, Zavaroni I: **The clinical reality of guidelines for primary prevention of cardiovascular disease in type 2 diabetes in Italy.** *Atherosclerosis* 2008, **198:**396-402.
41. Raum E, Lietzau S, Stegmaier C, Brenner H, Rothenbacher D: **For the majority of patients with diabetes blood pressure and lipid management is not in line with recommendations. Results from a large population-based cohort in Germany.** *Pharmacoepidemiol Drug Saf* 2008, **17:**485-494.
42. Wan Q, Harris MF, Jayasinghe UW, Flack J, Georgiou A, Penn DL, Burns JR: **Quality of diabetes care and coronary heart disease absolute risk in patients with type 2 diabetes mellitus in Australian general practice.** *Qual Saf Health Care* 2006, **15:**131-135.
43. Colhoun HM, Dong W, Barakat MT, Mather HM, Poulter NR: **The scope for cardiovascular disease risk factor intervention among people with diabetes mellitus in England: a population-based analysis from the Health Surveys for England 1991-94.** *Diabet Med* 1999, **16:**35-40.
44. Shojania KG, Ranji SR, McDonald KM, Grimshaw JM, Sundaram V, Rushakoff RJ, Owens DK: **Effects of quality improvement strategies for type 2 diabetes on glycemic control: a meta-regression analysis.** *JAMA: Journal of the American Medical Association* 2006, **296:**427-440.
45. Grimshaw JM, Thomas RE, MacLennan G, Fraser C, Ramsay CR, Vale L, Whitty P, Eccles MP, Matowe L, Shirran L, Wensing M, Dijkstra R, Donaldson C: **Effectiveness and efficiency of guideline dissemination and implementation strategies.** *Health Technol Assess* 2004, **8:**1-72.
46. Farmer AP, Légaré F, Turcor L, Grimshaw J, Harvey E, McGowan JL, Wolf F: **Printed educational materials: effects on professional practice and health care outcomes.** *Cochrane Database of Systematic Reviews* 2008:CD004398.
47. Zwarenstein M, Hux JE, Kelsall D, Paterson M, Grimshaw J, Davis D, Laupacis A, Evans M, Austin PC, Slaughter PM, Shiller SK, Croxford R, Tu K: **The Ontario printed educational message (OPEM) trial to narrow the evidence-practice gap with respect to prescribing practices of general and family physicians: a cluster randomized controlled trial, targeting the care of individuals with diabetes and hypertension in Ontario, Canada.** *Implement Sci* 2007, **2:**37.

doi: 10.1186/1745-6215-11-44
Cite this article as: Shah *et al.*, Evaluation of a toolkit to improve cardiovascular disease screening and treatment for people with type 2 diabetes: protocol for a cluster-randomized pragmatic trial *Trials* 2010, **11**:44
