# Supplementary material for: Effect of an Educational Toolkit on Quality of Care: A Pragmatic Cluster Randomized Trial
Source: PLoS Med. 2014 Feb 4;11(2):e1001588. doi: 10.1371/journal.pmed.1001588 (PMC3913553; doi:10.1371/journal.pmed.1001588)
Supplement: Text S2 — Trial protocol for the administrative data study. (DOC) [file pmed.1001588.s003.doc]

| Dataset Creation and Analytical Plan | | |
| --- | --- | --- |
| **Name and Number of Study** | | Evaluation of a toolkit to improve cardiovascular disease screening and treatment for people with type 2 diabetes (251.278) — population-based analysis |
| **Research Program** | | CDP |
| **Contacts** | | Baiju Shah, Merrick Zwarenstein |
| **PIA Approved?** | | Yes |
| **Short Description of Research Question** | | Does a guideline-based knowledge translation toolkit improve cardiovascular disease screening and treatment for patients with type 2 diabetes? |
| **List of Datasets Used** | | RPDB, ODD, HYPERTENSION, CIHI-DAD, OHIP, ODB, IPDB, CAPE, CCRS_LTC, diabetes_physnums.sas7bdat |
| Defining the Cohort | | |
| **Index Event** | Alive on 1 July 2009 with prevalent diabetes | |
| **Exclusions**  **(In order)** | Age ≤ 39  Residing in long-term care  Cannot be linked to a practice (see below) | |
| Variable Definitions | | |
| **Main Exposure or Risk Factor** | Assign each patient to a practice (see below). | |
| **Baseline Characteristics** | Patient level:  Sex  Age  Diabetes duration (categorize as <2 years, 2 to <5 years, 5 to <10 years, 10+ years)  Hypertension  Hospitalization for CVD (unstable angina, acute myocardial infarction or coronary revascularization) between 1st July 2004 and 30th June 2009  ECG between 31st August 2008 and 30th June 2009  Stress test / nuclear imaging between 31st August 2008 and 30th June 2009  Coronary angiography between 31st August 2008 and 30th June 2009  Outpatient ambulatory cardiology or internal medicine visit between 31st August 2008 and 30th June 2009  For patients born 31st August 1943 or earlier, determine baseline prescriptions between 31st August 2008 and 30th June 2009:  At least 1 ACE/ARB  At least 1 antihypertensive agent class  At least 2 antihypertensive agent classes  At least 3 antihypertensive agent classes  At least 1 statin  At least 1 glucose-lowering drug  At least 1 insulin  At least 1 nitrate  Practice level:  Intervention versus control group(from diabetes_physnums)  Number of physicians in the practice  Rurality  Diabetes patient volume | |
| **Outcome Definitions** | All outcome measures are dichotomous.  Measure between 1st July 2009 and 30th April 2010 (inclusive).  ECG  ECG stress test / nuclear imaging  Coronary angiography  Coronary revascularization  Outpatient ambulatory cardiology or internal medicine visit  Hospitalization for acute myocardial infarction  Hospitalization for AMI or unstable angina  Hospitalization for stroke  Hospitalization for stroke or transient ischemic attack  Mortality  Mortality or hospitalization for AMI or stroke  Mortality or hospitalization for AMI, unstable angina, stroke or TIA  For patients born 31st August 1943 or earlier, outcome prescriptions:  At least 1 ACE/ARB  At least 1 antihypertensive agent class  At least 2 antihypertensive agent classes  At least 3 antihypertensive agent classes  At least 1 statin  At least 1 glucose-lowering drug  At least 1 insulin  At least 1 nitrate | |
| **Long-term care residents** | People with ADMDATE ≤ 1 July, 2009 in CCRS_LTC data | |
| **Assigning patient to practice** | 1. Practice of the physician to whom patient is rostered in the CAPE tables with strtcape ≤ 1 July 2009 and endcape ≥ 1 July 2009. If the physician is not on the table, exclude the patient.  2. If the patient was not rostered, look for these OHIP feecodes between 1st July 2007 and 30th June 2009: A001, A003, A007, A903, E075, G212, G271, G372, G373, G365, G538, G539, G590, G591, K005, K013, K017, P004, where spec=’00’. Sum totpaid for those codes per practice. Assign patient to the practice with highest sum of costs. In case of ties, assign to the practice from which the last claim was made.  3. If those codes were not billed, exclude the patient. | |
| **Cardiology or internal medicine visit** | OHIP claim where IPDB main specialty = “CARDIOLOGY” or “INTERNAL MEDICINE”. Restrict to location=”OFFICE” in %ohip_location | |
| **Diabetes patient volume** | For each physician in the practice, extract all OHIP claims between 1 July 2009 and 30 April 2010 for patients in the ODD where servdate >= diagdate. Limit to location=’OFFICE’ from %ohip_location. Count the number of unique patients in the practice, divided by the number of doctors in the practice. Categorize as <100, 100 to <200, 200+. | |
| **Rurality** | Rurality Index of Ontario score for practice’s postal code: Major urban (0–9), non-major urban (10–44), rural (≥45) | |
| **Hypertension** | Prevalent hypertension in HYPERTENSION database as of 1 July 2009 | |
| **ECG** | OHIP feecodes G310, G313 | |
| **ECG stress test / nuclear** | OHIP feecodes G315, G319, G174, G111, G112, *or* OHIP feecodes G808 and G809 on the same date, *or* OHIP feecodes G608 and G609 on the same date. | |
| **Coronary angiography** | OHIP feecodes G297, G509, Z442 | |
| **Coronary revasc** | incodes 1IJ50, 1IJ57, 1IJ76, 1IJ80. Include SDS. | |
| **Acute MI** | dx10codes I21, I22. Inclsuspect=F. Dxtype=’M’ ’1’ ’2’ ’W’ ’X’ ’Y’ | |
| **Unstable angina** | dx10code I200. Inclsuspect=F. Dxtype=’M’ ’1’ ’2’ ’W’ ’X’ ’Y’ | |
| **Stroke** | dx10codes I61, I63, I64. Inclsuspect=F. Dxtype=’M’ ’1’ ’2’ ’W’ ’X’ ’Y’ | |
| **Transient ischemic attack** | dx10codes G450, G451, G452, G453, G458, G459. Inclsuspect=F. Dxtype=’M’ ’1’ ’2’ ’W’ ’X’ ’Y’ | |
| **ACE/ARB** | subclnam = ‘ACE INHIBITORS’ or ‘ACE INHIBITORS COMBINATION’ or ‘ANGIOTENSIN II ANTAGONIST’ or ‘ANGIOTENSIN II COMBINATION’ | |
| **Anti-hypertensive classes** | In all cases, include only tablet, capsule or kit forms, not IV, ophthalmic, topical, etc.  Note classes are not mutually exclusive, as some combination tablets appear under two different classes, and should be counted in each class.  ACE / ARB: as above  Alpha-blocker: drugname =: ‘DOXAZOSIN’ ‘PRAZOSIN’ or ‘TERAZOSIN’  Beta-blocker: subclnam=‘BETA-BLOCKERS’ or ‘BETA-BLOCKERS COMBINATION’ or drugname= ‘TIMOLOL MALEATE & HYDROCHLOROTHIAZIDE’, exclude drugname =: ‘SOTALOL’  Calcium-blocker: subclnam = ‘CALCIUM BLOCKERS’ or ‘CALCIUM BLOCKERS ANTILIPEMIC COMBINATIONS’ or drugname=’FELODIPINE & METOPROLOL’  Centrally acting: drugname =: ‘CLONIDINE’ ‘METHYLDOPA’ or ‘RESERPINE’  Diuretic: subclnam=’DIURETICS’ ‘DIURETICS (POTASSIUM-SPARING)’ ’ACE INHIBITORS COMBINATION ‘ or ‘ANGIOTENSIN II COMBINATION’ or ‘BETA-BLOCKERS COMBINATION’, or drugname= ‘LISINOPRIL & HYDROCHLOROTHIAZIDE’ ’EPROSARTAN MESYLATE & HYDROCHLOROTHIAZIDE’, ‘METHYLDOPA & HYDROCHLOROTHIAZIDE’ or ‘RESERPINE & HYDROCHLOROTHIAZIDE’, excluding drugname =: ‘BUMETANIDE’ ‘EPLERENONE’ ‘ETHACRYNIC’ ‘FELODIPINE & METROPROLOL’ ‘FUROSEMIDE’ ‘METOLAZONE’  Guanethidine: drugname =: ‘DEBRISOQUINE’ ‘GUANETHIDINE’  Hydralazine: drugname =: ‘HYDRALAZINE’  Minoxidil: drugname =: ‘MINOXIDIL’  Phenoxybenzamine: drugname =: ‘PHENOXYBENZAMINE’ | |
| **Statin** | subclnam="ANTILIPEMIC: STATINS" or "CALCIUM BLOCKERS ANTILIPEMIC COMBINATIONS” | |
| **Glucose-lowering drug** | subclnam=”INSULINS" or "ORAL ANTI-GLYCEMICS” | |
| **Insulin** | subclnam=”INSULINS" | |
| **Nitrate** | drugname = "ISOSORBIDE DINITRATE" or "NITROGLYCERIN" | |
| **Number of physicians in practice** | Count the number of unique cpso_ids per groupnum from diabetes_physnum data. | |
| Outline of Analysis Plan | | |
| Report baseline characteristics (at patient and practice levels) by intervention versus control group.  Calculate OR for each outcome, using generalized estimating equations to account for clustering of patients within practices. Correlation matrix – exchangeable. Adjust for sex, age, diabetes duration and prior CVD as patient-level variables; and intervention versus control and diabetes patient volume as practice-level variables. For the drug prescription outcomes among seniors, restrict to those patients who did not already achieve the outcome in the baseline period (i.e., look at statin outcome only among those patients not already on a statin at baseline). | | |
